# Comparative Analysis of Ventilatory Responses during Stress Tests in Patients with Chronic Pain: Implications for Therapeutic Interventions

**DOI:** 10.3390/jfmk9030122

**Published:** 2024-07-07

**Authors:** Patricio Barría, María Gaitán-Padilla, Daniel Gomez-Vargas, Gonzalo Cardenas Ampuero, Kay Gitterman, Bessie Cordova, Camilo A. R. Diaz, Flavio Roberti

**Affiliations:** 1 Rehabilitation Center Club de Leones Cruz del Sur, Punta Arenas 02281, Chile; pbarria@rehabilitamos.org (P.B.); gcardenas@rehabilitamos.org (G.C.A.); kay.gittermann@umag.cl (K.G.); bessie.cordova@rehabilitamos.org (B.C.); 2 Telecommunications Laboratory (Labtel), Electrical Engineering Department, Federal University of Espírito Santo, Vitória 29047-105, Brazil; maria.padilla@edu.ufes.br (M.G.-P.); camilo.diaz@ufes.br (C.A.R.D.); 3 Institute of Automatics, National University of San Juan, San Juan 5400, Argentina; dgomez@inaut.unsj.edu.ar; 4 Clinical Hospital Dr. Lautaro Navarro Avaria, Punta Arenas 01364, Chile

**Keywords:** fibromyalgia, chronic pain, ventilatory response, physical therapy, respiratory stress test

## Abstract

Understanding the differences in ventilatory responses during exercise between patients with fibromyalgia and those with other chronic pain disorders is crucial for developing effective therapeutic interventions, especially in exercise to identify the better physical therapy prescription. Both populations face unique challenges that impact their ability to engage in physical activity; yet, the underlying physiological responses can vary significantly. In this context, the methodology of this study entailed conducting a comparative analysis of the ventilatory response during exercise in patients with fibromyalgia and those with other chronic pain disorders. The experimental protocol included a total of 31 participants (n = 13 diagnosed with fibromyalgia and n = 18 diagnosed with other chronic pain conditions). All participants completed a stress test, where the ventilatory parameters were measured in three stages (i.e., resting, incremental exercise, and recovery). The results revealed significant differences (p<0.05) in ventilatory responses between both groups. Patients with fibromyalgia exhibited reduced time for the aerobic threshold and a higher respiratory frequency in the anaerobic threshold compared to those with other chronic pain disorders. Furthermore, fibromyalgia patients demonstrated higher values in the ventilatory coefficient during the test and in the recovery stage. In conclusion, these differences underscore the need for tailored exercise programs that specifically address the unique ventilatory challenges faced by fibromyalgia patients to improve their physical function and overall quality of life.

## 1. Introduction

Chronic pain is an unpleasant sensory and emotional experience that persists for at least three months [1]. This condition is multifactorial, where biological, psychological, and social factors contribute to the pain syndrome [2,3]. The International Classification of Diseases in the last revision (ICD-11) classifies this condition into chronic primary pain (CPC) and other secondary chronic pain syndromes [4]. Specifically, CPC affects one or more anatomical regions and often leads to emotional distress and functional disorders. In this way, CPC significantly impacts activities of daily living (ADLs) and social interactions [2].

Fibromyalgia (FM) is a CPC characterized by musculoskeletal pain, including muscular and articular stiffness, insomnia, fatigue, and mood and cognitive disturbances [5]. People diagnosed with FM exhibit high scores in neuropathic pain questionnaires, alterations in heat and cold detection thresholds, and altered pain responses [6]. These effects are related to dysfunctions in the immune system, resulting in an elevated inflammatory state and altered nociceptor sensitivity [7].

In general terms, chronic pain impairs between one-fifth and one-half of the worldwide population [8] and is linked to the leading causes of disability [9]. Statistically, pain accounts for 22% to 50% of medical consultations, where patients who exhibit chronic pain are 1.5 times more likely to visit healthcare services than healthy people [10]. Moreover, studies report an incidence rate of FM between 2% and 8%, with a higher prevalence in women [11].

Currently, strategies used to counteract pain and associated symptoms include pharmacological interventions, psychological support, adaptations to lifestyle, and physical therapy [12]. These approaches are designed to alleviate symptoms and improve patients’ quality of life. Pharmacological treatments typically involve medications such as anti-inflammatory drugs, opioids, and antidepressants, which target pain pathways and help reduce pain perception [13]. Physical therapy, on the other hand, focuses on improving mobility, reducing pain, and enhancing strength and functionality through exercises, manual therapy, and techniques like hydrotherapy [14]. In the case of psychological interventions, the aim is to address the symptoms related to the emotional and mental health of chronic pain patients [15]. Examples of physical therapy include stretching and strengthening exercises, massage, and joint mobilization [14]. Additionally, alternative methods such as electrotherapy have gained popularity. Electrotherapy methods include Transcutaneous Electrical Nerve Stimulation (TENS), which uses electrical currents to provide pain relief, and deep oscillation therapy [16], which uses low-frequency electrostatic impulses to reduce pain and inflammation. These diverse treatment modalities allow for a comprehensive approach to managing chronic pain, addressing both the physical and psychological aspects of the condition. Combined treatments, particularly pharmacological and non-pharmacological, represent a promising new strategy for chronic pain management in several pain syndromes [17]. Recent studies have demonstrated the efficacy of this approach in improving patient outcomes and quality of life. For example, Deodato et al. found that a combination of medication with Onabotulinumtoxin-A and physical therapy significantly reduced pain intensity and disability of participants with chronic migraine [18]. Similarly, Puentedura et al. reported enhanced pain relief and functional improvement in chronic lower back pain patients through the integration of physical therapy and pain awareness through neuroscience education [19]. These findings underscore the potential of combined treatment modalities in addressing complex pain conditions while also considering psychological interventions such as Cognitive Behavioral Therapy (CBT), pharmacological treatment tailored to chronic pain conditions, and interdisciplinary collaboration [13].

Notwithstanding, although strategies have achieved positive results, individuals with chronic pain often experience significant challenges in maintaining physical activity due to pain, muscle stiffness, and more [20]. Moreover, therapy strategies are currently applied in the same manner to both fibromyalgia and other chronic pain patients, even when a tailored and individual plan is generated by physiotherapists. A comparative analysis to determine whether it is viable to use different strategies is needed.

In this sense, understanding muscular behavior during exercise in people with chronic pain is crucial for developing effective exercise regimens. Physical activity is widely recognized as a beneficial intervention for managing fibromyalgia and other chronic pain disorders. However, the response to physical activity in these populations can be markedly different compared to those without the conditions [21]. Specifically, muscular behavior in individuals with fibromyalgia is often altered, with studies indicating increased muscle tenderness, slower muscle recovery, and a heightened perception of pain during and after exercise. These factors can lead to a reluctance to engage in physical activity, further exacerbating deconditioning and muscle weakness [22]. Furthermore, there is currently a lack of respiratory analysis specifically focused on chronic pain patients.

In this context, this study is designed to analyze the ventilatory responses during stress tests in two groups—patients with chronic pain and those with fibromyalgia—by comparing their ventilatory responses during maximum exercise tests. Thus, this work seeks to determine potential differences in their physiological responses when FM and OCP patients perform physical activity, with the hypothesis that the ventilatory responses would vary between both groups due to the specific causes and nature of fibromyalgia.

## 2. Materials and Methods

This is a comparative and observational study that aims to identify potential differences that could inform therapeutic interventions for FM and OCP conditions.

### 2.1. Participants

This study recruited 31 active female patients undergoing therapy at the Rehabilitation Center Club Leones Cruz del Sur who fulfilled the inclusion and exclusion criteria outlined below.

- Inclusion Criteria:
  Patients with musculoskeletal dysfunctions who have been diagnosed with either fibromyalgia (FM) or other chronic pain (OCP) disorders such as osteoarthritis, scoliosis, arthritis, and more. Patients with chronic fatigue were also considered for this study.
- Exclusion Criteria:
  – Pregnant women.
  – People with acute-stage cancer.
  – People with amputated lower limbs.
  – People who exhibit moderate or severe dependency.
  – People with sense alterations and cognitive impairments that impede participation in the stress test.

The sample size consisted of patients with chronic pain from the Magallanes region who attended the Rehabilitation Center to receive therapies. This study included patients who took symptomatic or prophylactic drugs. The use of medications was not suspended before or during the experiment.

Table 1 summarizes the information about the patients involved in this experiment.

### 2.2. Experimental Setup and Procedure

**Figure 1:**
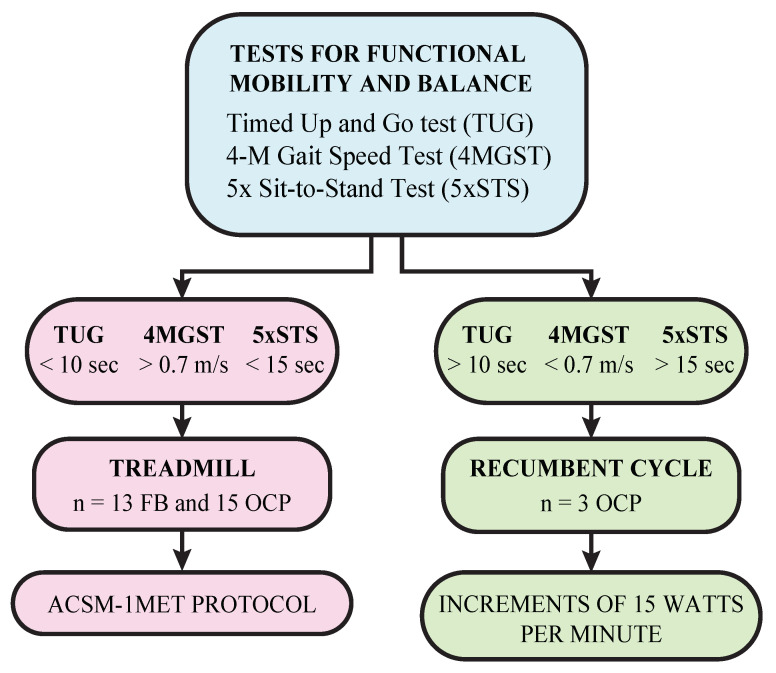
Methodology used to assign ergometer groups in the stress test. The test ranges represent the criteria used to assign each ergometer. The n values denote the number of patients with fibromyalgia (FM) and other chronic pain (OCP) in both groups.

This study, conducted during the last three months of 2023, included tests for functional mobility and balance to (1) measure the subject’s physical condition and (2) designate the ergometer used during the experiment—either a treadmill or a recumbent cycle. Participants underwent the Timed Up-and-Go (TUG) test, the 4-Meter Gait Speed Test (4MGST), and the 5X Sit-to-Stand (5xSTS) test, with the ergometer assigned using the methodology exhibited in Figure 1. Specifically, the TUG test assessed mobility, equilibrium, and fall risk [23,24,25,26]. The 5xSTS test measured transfer ability and provided a method to quantify lower-limb functional strength [27,28,29], and the 4MGST evaluated functional mobility [30,31].

Each participant was instrumented with a gas analyzer system and then completed one trial divided into three stages: (1) resting, (2) incremental exercise, and (3) recovery. In the resting stage, the gas analyzer system recorded the participant’s respiratory variables for 3 min. Subsequently, the subject started the exercise with the selected ergometer, increasing the intensity as follows:

- Treadmill: Step-wise with increments of 1 MET per minute.
- Recumbent Cycle: Incremental ramp of 15 watts per minute

**Figure 2:**
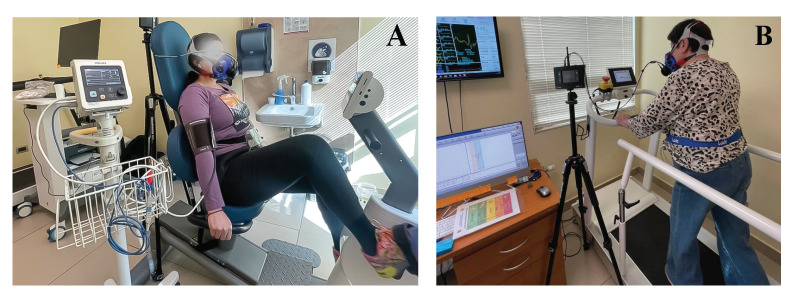
Experimental setup for this study: The left image (**A**) illustrates the protocol using the recumbent cycle, whereas the right image (**B**) depicts the exercise conducted on a treadmill.

The incremental exercise was conducted until the participant reached maximum effort or decided to discontinue. Finally, the protocol included a 3-min recovery stage, where the participant remained in the room until the ventilatory equilibrium returned to normal. Figure 2 illustrates the setup used in this experiment for each ergometer.

### 2.3. Ethical Approval

This study was approved by the institutional review committee of the Rehabilitation Center Club de Leones Cruz del Sur (Approval Code: CRCS_UID_020223), ensuring compliance with ethical and methodological standards. Participants signed informed consent prior to their participation.

### 2.4. Data Processing and Analysis

Firstly, data analysis included a filter stage to smooth the recorded signals every 15 steps and discard invalid steps. Subsequently, oxygen consumption (VO2), carbon dioxide production (VCO2), ventilatory coefficient (RER), tidal ventilation (VT), and ventilation per minute (VE) were used to estimate the participants’ ventilatory responses.

Considering the experimental procedure stages, this study measured RER, VO2, and VCO2 throughout the entire trial (i.e., resting, incremental exercise, and recovery). Additionally, it recorded the time to reach the aerobic threshold (T aerobic), anaerobic threshold (T anaerobic), and maximum effort (T maximum), as well as the respiratory frequency (RF) during the incremental exercise stage.

Statistically, the Shapiro–Wilk test was used to determine whether the data were normally distributed (*p* > 0.05). Therefore, based on the data distribution, a *t*-test (for normally distributed data) and a Wilcoxon test (for non-normally distributed data) were used to find statistical differences in the calculated parameters between FM and OCP across the trial.

### 2.5. Study Equipment

The experiment employed two ergometers to execute the maximum effort tests: (1) a treadmill (Valiant Rehab, LODE, Groningen, The Netherlands) and (2) a recumbent cycle ergometer (Corival Recumbent, LODE, The Netherlands). Moreover, a respiratory gas analyzer (K5 System, COSMED, Rome, Italy) measured the participant’s respiratory variables through the OMMIA Software (Version 2.3, COSMED, Italy). This study employed an Optiflex 7010 computer (i5-3470, DELL, Round Rock, TX, USA) for data acquisition and processing. Additionally, an ALL-IN-ONE 24-DP0158QE computer (i7-10510U, HP, Palo Alto, CA, USA) and RStudio software (Version 2024.04.2, Posit, Boston, MA, USA) were employed for statistical analysis.

## 3. Results

The results of the stress test were obtained across three stages (i.e., resting, incremental exercise, and recovery) from patients diagnosed with fibromyalgia (FM) and other chronic pain (OCP) disorders.

Table 2 presents the initial values for oxygen consumption (VO2), carbon dioxide production (VCO2), and ventilatory coefficient (RER) for both patient groups during the resting stage. In statistical terms, none of the measured variables showed significant differences (p>0.05) when comparing the FM group with the OCP group.

Concerning the incremental exercise stage, Table 3 summarizes the results for each group at three thresholds (i.e., maximum, aerobic, and anaerobic). The assessed parameters in this stage were time (T), ventilation per minute (VE), ventilatory threshold (VT), respiratory frequency (RF), VO2, VCO2, and RER.

**Figure 3:**
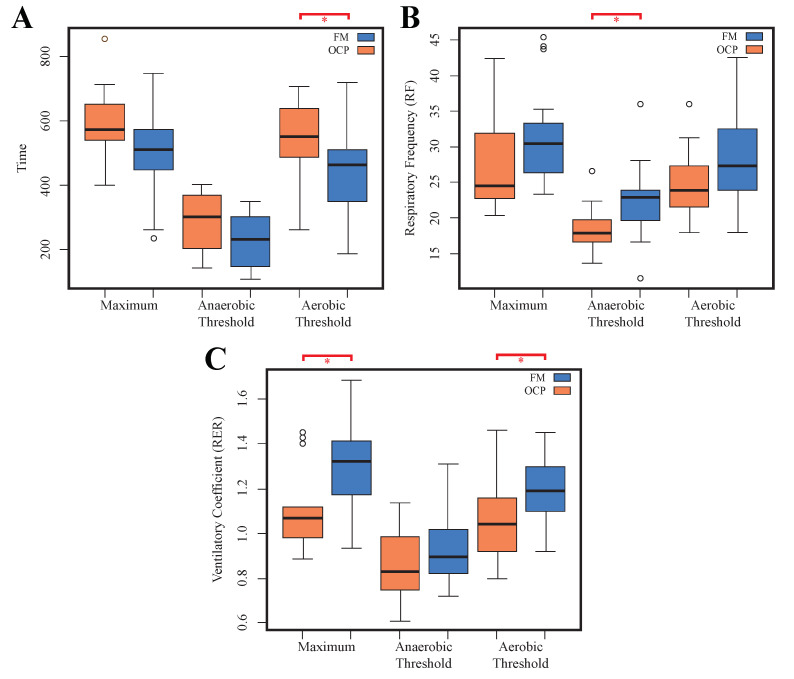
Distribution of time (**A**), respiratory frequency (**B**), and ventilatory coefficient (**C**) across maximum, anaerobic, and aerobic thresholds during the incremental exercise stage. Thresholds highlighted in red and with asterisk indicate statistically significant differences between the fibromyalgia and other chronic pain groups.

In this stage, four variables, i.e., T anaerobic (p=0.03), RF aerobic (p=0.02), RER maximum (p=0.01), and RER anaerobic (p=0.02), exhibited statistically significant differences between both groups. Specifically, the FM group demonstrated a significantly longer anaerobic time compared to the OCP group (see Figure 3A), suggesting potentially superior anaerobic endurance in fibromyalgia patients during exercise. The OCP group exhibited a higher aerobic RF during the exercise test (*p* < 0.05), indicating a distinct ventilatory response pattern under aerobic conditions between the two groups, as shown in Figure 3B. Finally, the maximum RER and anaerobic RER were significantly higher in the OCP group during the test (see Figure 3C ), resulting in differences in metabolic substrate utilization during exercise compared to the FM group.

Regarding the recovery stage, Table 4 summarizes the VO2, VCO2, and RER values obtained from both patient groups, encompassing measurements at three intervals (i.e., 1, 2, and 3 min after completing the incremental exercise stage).

**Figure 4:**
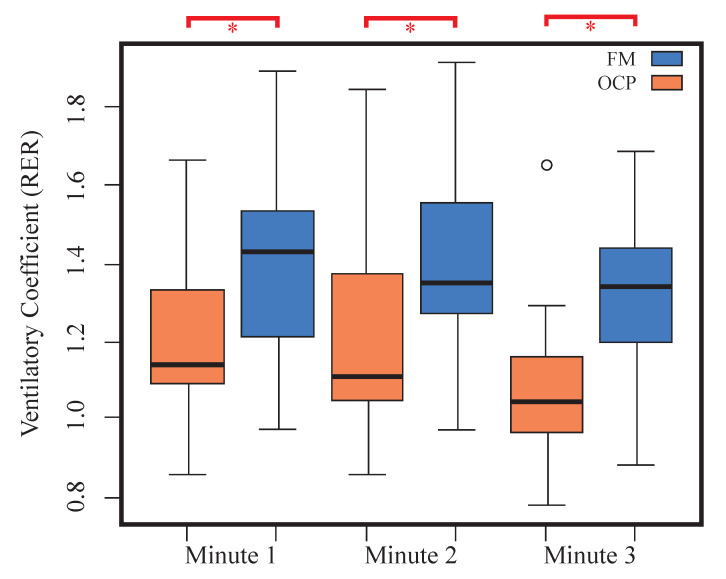
Distribution of the ventilatory coefficient for the recovery stage during three intervals. Intervals highlighted in red and with asterisk indicate statistically significant differences between the fibromyalgia and other chronic pain groups.

In statistical terms, RER exhibited significant differences between both groups throughout the recovery stage, with p-values of p=0.04 for the first minute, p=0.03 for the second minute, and p=0.01 for the third minute. Specifically, patients with other chronic pain disorders tended to increase the ventilatory coefficient compared to the FM group (see Figure 4). In this sense, both groups demonstrated distinct ventilatory recovery dynamics and ongoing metabolic processes, as well as differences in energy substrate utilization post-exercise.

**Figure 5:**
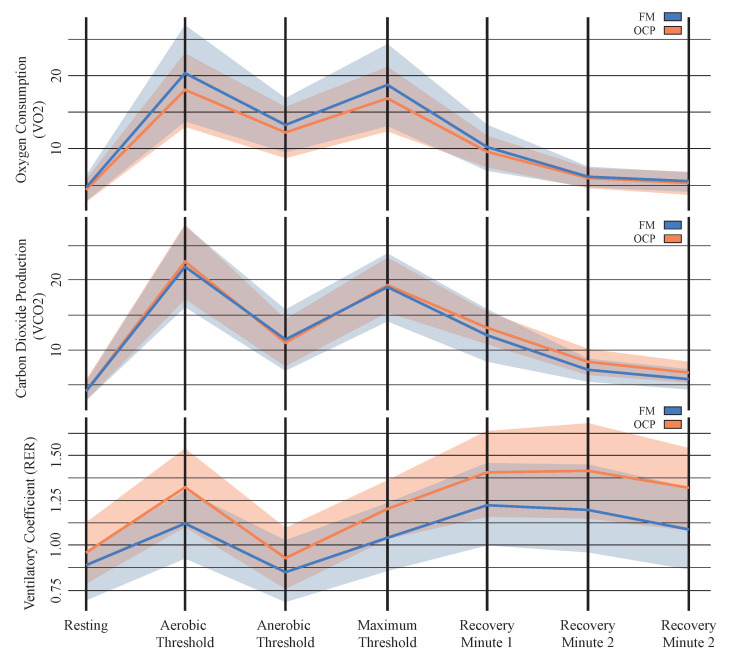
Oxygen consumption, carbon dioxide production, and ventilatory coefficient reported during the experiment for both patient groups. The lines represent the mean values, and the shaded areas indicate data dispersion.

Considering all stages in this experiment, Figure 5 shows the progression of VO2, VCO2, and RER for FM and OCP patients. On the one hand, patients with OCP exhibited increased oxygen consumption during the trial compared to the fibromyalgia group, although VCO2 demonstrated similar behavior for both groups. On the other hand, the ventilatory coefficient was higher in the OCP group than in patients with fibromyalgia.

## 4. Discussion

The finding that the fibromyalgia (FM) group demonstrated a significantly longer anaerobic time compared to the other chronic pain (OCP) group suggests potential differences in anaerobic metabolism and exercise tolerance between these patient groups. Anaerobic time describes the interval during which the body can sustain intense exercise before reaching a critical threshold. Therefore, aerobic metabolism cannot meet the increased energy demand, leading to reliance on anaerobic pathways [32]. Lehto et al. conducted a study to determine the differences in the cardiac and ventilatory variables during exercise with 35 patients diagnosed with FM and 23 women as the control group, concluding that the exercise responses were normal and not suggestive of muscle metabolism pathology. Moreover, the VO2 peak and cardiography impedance were lower in the FM group, indicating a possible influence of exercise on physiological responses [33].

In this sense, the altered muscle fiber type and endurance in fibromyalgia patients affect anaerobic metabolism, resulting in a higher proportion of slow-twitch muscle fibers. Thus, the FM group could be more fatigue-resistant and capable of sustaining aerobic metabolism for longer durations before transitioning to anaerobic energy production [34]. Additionally, the literature has reported differences in the mitochondrial function and oxidative capacity of muscle cells, which could have contributed to the observed anaerobic endurance differences [35,36]. Fibromyalgia patients might have enhanced mitochondrial efficiency, allowing for prolonged aerobic energy production before transitioning to anaerobic metabolism [33,37]. The anaerobic differences observed in this experiment could also be related to pain perception and tolerance, which are remarkable characteristics of FM. Hence, fibromyalgia patients might exhibit different pain modulation mechanisms that enable them to sustain anaerobic exercise for prolonged periods compared to individuals with other chronic pain disorders [38]. Rodríguez-Almagro et al. conducted a systematic review to evaluate the effect of physical exercise-based therapy on pain, the impact of the disease, quality of life, and anxiety in patients with FM to determine the effect of different modes of physical therapy and the most effective dose of exercise for improving each outcome [39]. In their results, among patients with FM, exercise was effective at reducing pain, the impact of the disease, and anxiety, as well as increasing QoL, showing the importance of evaluating the physiological responses of patients with chronic pain to exercise.

On the other hand, the results revealed that individuals with other chronic pain disorders exhibited higher aerobic respiratory frequency (RF) compared to fibromyalgia patients during the incremental exercise test. This outcome could be related to the ventilatory control and perception of dyspnea. Patients with fibromyalgia may exhibit altered respiratory control patterns or heightened sensitivity to dyspnea, resulting in lower RF during aerobic exercise [40]. In this sense, differences in ventilatory control mechanisms and perception of dyspnea (i.e., shortness of breath) might contribute to variations in RF between patients with FM and OCP disorders.

Along the same line, muscle metabolism and oxygen utilization could also cause differences in RF between both groups. Fibromyalgia patients may exhibit different patterns of muscle oxygenation and utilization during aerobic exercise, affecting the respiratory response [41]. On the other hand, cardiovascular adaptations to exercise, such as heart rate response, could also impact respiratory frequency. Individuals with other chronic pain disorders usually exhibit unique cardiovascular responses, which require a higher RF to maintain oxygen delivery and remove carbon dioxide during aerobic exertion [42].

This study found that the maximum and anaerobic ventilatory coefficient (RER) values were significantly higher in the OCP group than in the FM group during the exercise test. The higher RER values in the OCP group suggest increased reliance on carbohydrate metabolism during exercise, potentially reflecting differences in metabolic efficiency and substrate utilization compared to the FM group. This outcome could be attributed to factors such as altered muscle metabolism or insulin resistance, which are commonly observed in chronic pain conditions [34].

Likewise, differences in exercise intensity and energy expenditure between OCP and FM patients could also contribute to variations in RER. Patients with OCP might reach higher exercise intensities, resulting in greater reliance on anaerobic metabolism and thus elevated RER values [43,44]. Furthermore, some differences in muscle fatigue and lactate production during exercise could also influence RER. A higher RER in OCP patients might indicate an earlier onset of anaerobic metabolism and lactate accumulation, potentially due to altered muscle physiology or pain-related neuromuscular adaptations [44].

The outcomes during the recovery stage revealed that the RER values at 1, 2, and 3 min of recovery were significantly higher in the OCP group than in patients with FM. These differences are associated with delayed metabolic recovery. Specifically, the higher RER values observed in the OCP group indicate prolonged reliance on anaerobic metabolism post-exercise, contributing to slower metabolic recovery. This behavior could be attributed to factors such as impaired muscle recovery processes, altered autonomic function, or persistent pain-related stress responses [45]. In addition, chronic pain conditions may disrupt metabolic homeostasis and contribute to heightened stress responses. The elevated RER during recovery in the OCP group could reflect a prolonged recovery phase characterized by metabolic imbalances and persistent physiological stress [44]. These results influence post-exercise RER, showing that OCP patients might exhibit reduced aerobic capacity, leading to sustained reliance on anaerobic metabolism during the recovery period [35].

In this context, understanding the anaerobic time differences between fibromyalgia and other chronic pain disorders can have practical implications for exercise prescription and management strategies. Firstly, studies have demonstrated the importance of correct exercise prescriptions for these conditions [46]. Personalized exercise programs should consider individual anaerobic thresholds and endurance capacities to optimize benefits and minimize discomfort during physical activity. Also, these results can lead to adopting methods for symptom management. Insights into anaerobic metabolism can inform symptom management approaches. For instance, exercise interventions focusing on improving anaerobic capacity may be particularly beneficial for individuals with fibromyalgia.

Considering the differences in aerobic RF and RER during tests and recovery between FM and OCP patients, exercise intensity monitoring and breathing techniques could be incorporated into physical therapy. Monitoring RF can guide exercise intensity prescription to ensure that physical therapy is tailored to the patient’s needs. Patients with fibromyalgia could benefit from exercise programs that take into account their lower aerobic RF. These programs should focus on pacing strategies to optimize aerobic conditioning while minimizing the risk of excessive dyspnea. Incorporating breathing techniques into exercise interventions can mitigate discomfort and optimize respiratory efficiency during aerobic activities for patients with chronic pain disorders. Additionally, monitoring RER during recovery stages can aid in personalizing rehabilitation interventions to address metabolic dysregulation and optimize recovery. This approach can enhance overall exercise tolerance and improve functional outcomes in individuals with chronic pain disorders.

It is worth noting that there are currently no studies in the literature directly comparing ventilatory responses during exercise between patients diagnosed with fibromyalgia and those with other chronic pain disorders. This study addresses this gap by conducting a comparative analysis focused on ventilatory parameters in these distinct patient populations.

This study aimed to compare the ventilatory responses in fibromyalgia and other chronic pain disorders to determine the effectiveness of combined and personalized treatments. While the findings provide valuable insights into ventilatory responses during stress tests in patients with chronic pain and fibromyalgia, it is important to acknowledge several limitations. Firstly, the small sample size of 31 participants, with 13 individuals diagnosed with fibromyalgia and 18 with other chronic pain issues, may limit the generalizability of the results. This small sample size could potentially affect the statistical power to detect differences between groups and was considered when interpreting the findings. Despite these limitations, this study contributes to the understanding of ventilatory responses in chronic pain populations and informs future research directions in therapeutic interventions.

This is an optimal approach for ventilatory evaluation of the chronic pain population, especially in Chile. Future work will include the evaluation of more patients and the development of personalized therapy plans for pre- and post-therapy assessment using the maximum effort test to analyze the ventilatory response after exercise prescription based on specific ventilatory models. Also, this analysis can be used to guide exercise attempt decisions in subjects with fibromyalgia and other chronic pain conditions by providing insight into ventilatory patterns during exercise, as well as aerobic and anaerobic thresholds.

## 5. Conclusions

This study provides insights into ventilatory responses observed during maximal exercise and recovery among patients with fibromyalgia (FM) and other chronic pain (OCP) disorders. Fibromyalgia patients demonstrated enhanced anaerobic endurance, while individuals with other chronic pain disorders exhibited unique patterns of aerobic respiratory frequency and metabolic utilization. These findings contribute to a deeper understanding of respiratory adaptations in different chronic pain conditions, highlighting the need for tailored exercise interventions and symptom management strategies. Further investigation is needed to elucidate the underlying mechanisms driving differences in anaerobic endurance between fibromyalgia and other chronic pain disorders. Longitudinal studies assessing changes in anaerobic capacity over time and exploring the impact of specific exercise interventions could improve the respiratory response to exercise, optimizing therapeutic approaches for chronic pain conditions.

Additionally, future research should aim to clarify the physiological mechanisms supporting differences in RER between FM and OCP patients, investigating the impact of exercise interventions on metabolic responses and substrate utilization patterns. Lastly, the higher recovery RER observed in individuals with other chronic pain disorders compared to fibromyalgia patients suggests prolonged metabolic stress and delayed recovery kinetics post-exercise. Ongoing research efforts aimed at understanding and addressing respiratory and metabolic adaptations in fibromyalgia and other chronic pain disorders will contribute to the development of evidence-based therapeutic approaches tailored to individual patient needs. These results and analyses can enhance treatment efficacy and improve the quality of life for individuals who exhibit chronic pain.

## Figures and Tables

**Table 1:** Characteristics of enrolled participants.

	Fibromyalgia (FM)	Other Chronic Pain (OCP)
Number of participants	13	18
Age (years)	53 ± 7	57 ± 12
Weight (kg)	70 ± 12	74 ± 16
Height (cm)	154 ± 5	156 ± 8
Body mass index	29 ± 5	30 ± 7
Visual analogue scale (0–10)	7 ± 1	6 ± 2
Time living with pain (years)	9 ± 5	5 ± 5

**Table 2:** Median and interquartile range (IQR) of variables measured during the resting exercise stage for patients diagnosed with fibromyalgia and other chronic pain disorders. Highlighted values denote non-normal data distribution, i.e., p<0.05 for the Shapiro–Wilk test. The abbreviations refer to oxygen consumption (VO2), carbon dioxide production (VCO2), and ventilatory coefficient (RER).

	Fibromyalgia (FM)		Other Chronic Pain (OCP) Disorders	
Measured Variable	Median	(IQR)	Median	(IQR)
VO2	4.2	(3.1–6.0)	4.0	(3.1–4.5)
VCO2	3.8	(2.7–4.6)	3.8	(3.1–4.0)
RER	0.8	(0.8–1.0)	0.9	(0.9–1.0)

**Table 3:** Median and interquartile range (IQR) of variables measured during the incremental exercise stage. Highlighted values denote non-normal data distribution, i.e., p<0.05 for the Shapiro–Wilk test. Variables in bold and with an asterisk denote significant differences between both patient groups. The abbreviations refer to time (T), ventilatory threshold (VT), ventilation per minute (VE), respiratory frequency (RF), oxygen consumption (VO2), carbon dioxide production (VCO2), and ventilatory coefficient (RER).

		Fibromyalgia (FM)		Other Chronic Pain (OCP) Disorders	
Measured Variable		Median	(IQR)	Median	(IQR)
**T**	maximum	571.0	(539.0–651.0)	510.5	(460.3–572)
	aerobic	302.0	(203.0–368.0)	232.0	(147.8–302)
	anaerobic *	551.0	(488.0–639.0)	462.0	(350.0–506.8)
VE	maximum	37.6	(34.4–42.9)	43.4	(39.0–50.5)
	aerobic	19.5	(17.2–22.6)	22.3	(18.7–25.8)
	anaerobic	34.2	(30.8–36.5)	37.2	(31.2–43.0)
VT	maximum	1.4	(1.3–1.5)	1.4	(1.3–1.6)
	aerobic	0.9	(0.8–1.3)	0.9	(0.9–1.2)
	anaerobic	1.4	(1.3–1.6)	1.3	(1.2–1.5)
**RF**	maximum	24.6	(22.8–24.6)	30.4	(26.6–33.2)
	aerobic *	17.8	(16.7–19.8)	22.8	(20.0–23.8)
	anaerobic	23.8	(21.6–27.2)	27.3	(24.3-32.3)
VO2	maximum	22.9	(14.6–23.5)	17.8	(13.6–21.5)
	aerobic	14.0	(10.7–16.8)	12.1	(9.6–13.9)
	anaerobic	18.7	(14.4–22.7)	16.5	(13.4–20.2)
VCO2	maximum	21.9	(20.0–25.2)	22.6	(19.3–26.1)
	aerobic	10.6	(8.0–13.5)	11.5	(9.0–13)
	anaerobic	20.1	(18.1–21.3)	19.1	(17.0–22.1)
**RER**	maximum *	1.1	(1.0–1.1)	1.3	(1.2–1.4)
	aerobic	0.8	(0.8–1.0)	0.9	(0.8–1.0)
	anaerobic *	1.0	(0.9–1.2)	1.2	(1.1–1.3)

**Table 4:** Median and interquartile range (IQR) of variables measured during the recovery stage. Highlighted values denote non-normal data distribution estimated by the Shapiro–Wilk test (p<0.05). Variables in bold and with an asterisk denote significant differences between both patient groups. The abbreviations refer to oxygen consumption (VO2), carbon dioxide production (VCO2), and ventilatory coefficient (RER).

		Fibromyalgia (FM)		Other Chronic Pain (OCP) Disorders	
Measured Variable		Median	(IQR)	Median	(IQR)
VO2	1 min	10.1	(8.0–12.4)	9.5	(8.4–10.4)
	2 min	6.1	(5.0–6.9)	6.0	(5.3–6.6)
	3 min	5.5	(5.2–5.9)	5.2	(4.5–5.9)
VCO2	1min	11.5	(10.3–13.6)	13.2	(12.2–14.5)
	2 min	6.9	(6.1–8.0)	8.3	(7.2–9.1)
	3 min	5.4	(5.0–6.8)	6.8	(6.2–7.7)
**RER**	1 min *	1.1	(1.1–1.3)	1.4	(1.2–1.5)
	2 min *	1.1	(1.1–1.4 )	1.3	(1.3–1.5)
	3 min *	1.0	(1.0-1.2)	1.3	(1.2–1.4)

## Data Availability

Data are available on reasonable request by email to Patricio Barría (pbarria@rehabilitamos.org).

## Abbreviations

The following abbreviations are used in this manuscript:

CPC	Chronic Primary Pain
FM	Fibromyalgia
OCP	Other Chronic Pain
TUG	Timed Up and Go Test
4MGST	4-M Gait Speed Test
5xSTS	5X Sit-to-Stand Test
VO2	Oxygen Consumption
VCO2	Carbon Dioxide Production
RER	Ventilatory Coefficient
VT	Tidal Ventilation
VE	Ventilation per Minute
T	Time
RF	Respiratory Frequency
